# An Image Focusing Method for Sparsity-Driven Radar Imaging of Rotating Targets

**DOI:** 10.3390/s18061840

**Published:** 2018-06-05

**Authors:** Ngoc Hung Nguyen, Kutluyıl Doğançay, Hai-Tan Tran, Paul Berry

**Affiliations:** 1 School of Engineering, University of South Australia, Mawson Lakes, SA 5095, Australia; kutluyil.dogancay@unisa.edu.au; 2 National Security and ISR Division, Defence Science and Technology Group, Edinburgh, SA 5111, Australia; haitan.tran@dst.defence.gov.au (H.-T.T.); paul.berry@dst.defence.gov.au (P.B.)

**Keywords:** radar imaging, rotating target, sparsity, image focusing, high-resolution ISAR, sparse reconstruction, compressive sensing, micro-motion, micro-Doppler

## Abstract

This paper presents a new image focusing algorithm for sparsity-driven radar imaging of rotating targets. In the general formulation of off-grid scatterers, the sparse reconstruction algorithms may result in blurred and low-contrast images due to dictionary mismatch. Motivated by the natural clustering of atoms in the sparsity-based reconstructed images, the proposed algorithm first partitions the atoms into separate clusters, and then the true off-grid scatterers associated with each cluster are estimated. Being a post-processing technique, the proposed algorithm is computationally simple, while at the same time being capable of producing a sharp and correct-contrast image, and attaining a scatterer parameter estimation performance close to the Cramér–Rao lower bound. Numerical simulations are presented to corroborate the effectiveness of the proposed algorithm.

## 1. Introduction

Radar imaging of a rotating target, or more generally of targets with micro-motion, has recently received considerable interest thanks to its important applications in both civilian and military domains [1,2,3]. Micro-motion refers to the ‘in-place’ motion, as distinct from the bulk translational motion, which may include a target’s own rotation, or rotation (or vibration) of certain structural components of the target. Micro-motions and the resulting micro-Doppler modulations may be undesirable as they can interfere with other processing for the bulk target; or they may be exploited as an extra target signature for target recognition. The main focus of this paper is on a rigid-body rotational type of micro-motion, typical examples of which include helicopter rotor blades and propellers of fixed-wing aircraft. We are particularly interested in imaging such a rotating object with a narrowband microwave radar.

Sparse reconstruction and compressive sensing, a powerful framework for solving ill-posed linear inverse problems [4,5,6,7,8,9,10], has been applied to imaging of rotating targets [11,12,13,14,15,16]. A micro-Doppler parameter estimation technique based on parametric sparse representation and pruned orthogonal matching pursuit was presented in [11]. A sparsity-driven radar imaging technique for rotating blades was developed in [13,14] based on the orthogonal matching pursuit algorithm, while other greedy pursuit algorithms were considered in [15]. The use of various convex relaxation algorithms for radar imaging of rotating targets was studied in [16]. The work in [13,14,15] focuses on blade-like targets where the tilted-wire scatterer model is applicable. On the other hand, the point-scatterer model is considered in [11,16]. Compared to the tilted-wire scatterer model, which is particularly relevant for blade-like targets, the point-scatterer model can be applied to more general target shapes and thus is widely used in the literature. In the last few years, the point-scatterer model has been studied in the context of sparsity-driven inverse synthetic aperture radar (ISAR) imaging [17,18,19]. The framework of sparse reconstruction and compressive sensing has also been applied to various applications in the broader context of radar imaging (see, e.g., [20,21,22,23,24,25,26,27]).

Much of the current literature on sparsity-based radar imaging of rotating micro-motion targets assumes that the true scatterers that constitute the target are located on a grid of uniformly-spaced spatial points (i.e., “on-grid”) that make up the dictionary. However, in real-life applications, true scatterers are always off-grid. Off-grid problems have been known to cause significant degradation in the sparse reconstruction performance due to dictionary mismatch (see, e.g., [28,29,30]). As will be demonstrated in this paper, the mismatch between the scatterer positions and the dictionary grid can severely defocus the reconstructed image of a rotating target. In particular, a dictionary that is too coarse may lead to a completely distorted image, while a fine dictionary may cause image blurring.

One intuitive solution to counter off-grid effects is to increase the grid density. However, using denser grids not only increases the computational complexity, but also undesirably results in higher mutual coherence in the dictionary. Another solution to the off-grid problem is to consider the sparse reconstruction problem in continuous parameter space as in [31,32]. In particular, continuous basis pursuit was developed in [31] for the sparse decomposition problem of translation-invariant signals by using an alternative discrete basis that accounts explicitly for the continuous time-shifts in the signal. The work in [32] proposed an atomic norm minimization approach to estimate frequency components of a mixture of complex sinusoids from partially-observed time samples. However, these gridless methods are computationally expensive due to the requirement of having to solve semidefinite programs [33]. In addition, the gridless methods [31,32] are application-specific solutions and their extension to general parameter estimation problems is still an open research problem [33].

In contrast to [31,32], several other works have addressed the off-grid problem directly on the conventional discrete parameter space including dictionary perturbation [34,35,36,37], parameter perturbation [38,39], joint-sparse recovery [40] and sparse Bayesian learning [41,42,43]. In [35,36], total least-squares based solutions were proposed by perturbing the dictionary atoms. In [37], $l_{1}$ minimization based algorithms were developed to tackle a linear structured perturbation in the dictionary. A perturbed orthogonal matching pursuit algorithm was proposed in [34] by applying a controlled perturbation mechanism on the atoms selected by the algorithm. Different to [34,35,36,37], which perturb the dictionary matrix, the works in [38,39] aimed to perturb the grid parameters used to construct the dictionary atoms, resulting in the parameter perturbed orthogonal matching pursuit [38] and the adaptive matching pursuit with constrained total least-squares [39]. A joint-sparse recovery method was developed in [40] to overcome structured dictionary mismatches. On the other hand, sparse Bayesian learning-based algorithms [41,42,43] tackle the off-grid problems by exploiting the structure of the dictionary atoms. By jointly estimating the grid offset and performing sparse reconstruction, these techniques are capable of dealing with the off-grid problem. However, this, at the same time, introduces more unknown variables to be estimated and complicates the algorithm development [33].

In this paper, we propose a novel image focusing algorithm to focus the blurred sparsity-driven reconstructed images of rotating targets in the general case of off-grid scatterers. The proposed algorithm takes the defocused image given by any sparse reconstruction algorithm as the input and produces a focused image as the output. Being a post-processing technique, the main advantage of the proposed approach lies in its simplicity and low complexity while at the same time being capable of achieving scatterer parameter estimation performance close to the Cramér–Rao lower bound (CRLB). The proposed algorithm consists of two stages: (I) cluster analysis, and (II) joint estimation of scatterer position and coefficient. In Stage I, the dictionary atoms in the input image are partitioned into a number of clusters. The idea behind Stage I is motivated by the fact that each off-grid scatterer typically induces a group of dictionary atoms located in its vicinity as a result of dictionary mismatch. In Stage II, each of the clusters obtained from Stage I is replaced by an equivalent estimated scatterer. Since the scatterer positions and reflection coefficients are jointly estimated in a continuous parameter domain, more accurate estimates for the positions and reflectivities of true off-grid scatterers are obtained rather than using the dictionary grid. Consequently, the proposed algorithm can produce a sharp image of the target with a correct image contrast. The effectiveness of the proposed algorithm is demonstrated via numerical simulations. Moreover, the mean-squared errors of the estimates of the locations and reflection coefficients of the true scatterers obtained by the proposed algorithm are shown to be comparable to the CRLB derived from estimation theory.

The paper is organized as follows. Section 2 presents an overview of the problem of sparsity-driven radar imaging of a rotating micro-motion target. Section 3 analyzes the technical challenges associated with off-grid scatterers. The proposed focusing algorithm is presented in Section 4. Section 5 derives the CRLB for the estimates of the positions and reflection coefficients of true scatterers. Comparative simulation studies are presented in Section 6 and conclusions are drawn in Section 7.

## 2. Overview of Sparsity-Driven Radar Imaging of a Rotating Target

**Figure 1:**
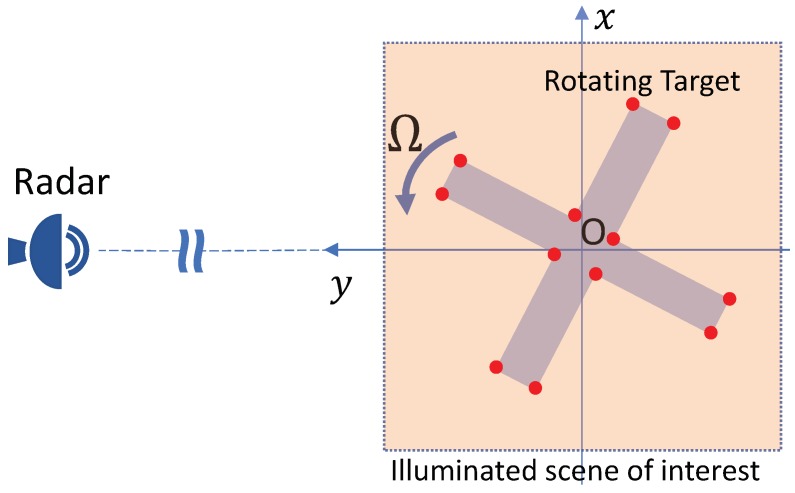
The radar-target geometry of the considered radar imaging problem of a rotating target.

We consider a two-dimensional (2D) radar imaging problem with a monostatic single-frequency continuous-wave radar located in the far field of a rotating micro-motion target as depicted in Figure 1. The target is modelled as a turnable object rotating around the rotation center *O*. Here, the origin of the coordinate system is placed in the rotation center of the target while the radar is located in the far field of the positive *y*-direction. In this paper, the target is modelled as a rigid ensemble of non-interacting point scatterers (the Born approximation), where the reflection coefficient of each scatterer is complex-valued with generally unknown amplitude and phase.

After translational motion compensation, the complex-valued energy-normalized baseband signal returned from a rotating point scatterer at the initial location $p={\left[x,y\right]}^{T}$ is given by [1,2]

(1) $$\vartheta \left(p,t\right)=A\exp\left\{ibr\sin(\Omega t+\psi )\right\},$$

where *A* is a normalization constant such that the signal energy over the coherent processing interval (CPI) is normalized to unity, b=4π/λ with λ denoting the radar wavelength, $r=\sqrt{x^{2}+y^{2}}$ and $\psi =\tan^{-1}\left\{y/x\right\}$ are the radius and angle of the scatterer, respectively, and Ω is the rotational velocity of the scatterer. Note that $\tan^{-1}\left\{\cdot \right\}$ denotes the 4-quadrant arctangent and $i^{2}=-1$. Here, the rotational velocity Ω is assumed to be constant over the CPI and known a priori. In many practical applications, Ω can be accurately estimated via relatively simple autocorrelation methods. In addition, we assume that translational motion compensation has been performed in a pre-processing step with negligible errors.

Assume that the illuminated target consists of scatterers located at cross-range position *x* and down-range position *y*, and with the complex reflection coefficient ρ(x,y). The continuous form of the total backscattered baseband signal is given by

(2) s(t)=∫∫ρ(p)ϑ(p,t)dxdy.

In radar imaging, it is common practice to discretize the reflection function ρ(p) over cross-range and down-range directions on a grid of points $p_{G,n}={\left[x_{G,n},y_{G,n}\right]}^{T}$ to form the reflection vector $\rho_{G}={\left[\cdots ,\rho_{G,n},\cdots \right]}_{n=1,\cdots ,N}^{T}$ and to sample the backscattered baseband signal at discrete time $t_{m}$ as $s_{m}=s\left(t_{m}\right)$ to form $s={\left[\cdots ,s_{m},\cdots \right]}_{m=1,\cdots ,M}^{T}$. This results in the discrete version of s(t) in Equation (2) given by

(3) $$s=\sum_{n=1}^{N}\rho_{G,n}\vartheta \left(p_{G,n}\right)=\Phi_{G}\rho_{G},$$

where

(4a) $$\begin{array}{cc} \Phi_{G} & ={\left[\cdots ,\vartheta \left(p_{G,n}\right),\cdots \right]}_{n=1,\cdots ,N}, \end{array}$$

(4b) $$\begin{array}{cc} \vartheta (p) & ={\left[\cdots ,\vartheta \left(p,t_{m}\right),\cdots \right]}_{m=1,\cdots ,M}^{T}. \end{array}$$

Note that ϑ(p) is the discrete version of ϑ(p,t) in Equation (1) with $A=1/\sqrt{M}$. In the context of sparsity and compressive sensing, $\Phi_{G}$ is commonly referred to as the overcomplete dictionary matrix and its columns $\vartheta \left(p_{G,n}\right)$ are referred to as atoms, each corresponding to the energy-normalized signal returned from a hypothetical scatterer located at a grid point on the reflectivity map.

In practice with the presence of noise, the noisy backscattered radar signal is given by

(5) $$\tilde{s}=s\left(\rho_{G}\right)+e=\Phi_{G}\rho_{G}+e,$$

where $e={\left[\cdots ,e(t_{m}),\cdots \right]}_{m=1,\cdots ,M}^{T}$. In this paper, e(t) is assumed to be circularly-symmetric complex Gaussian noise arising from the thermal noise in the radar hardware with variance $\sigma^{2}={E\{|e\left(t\right)|}^{2}\}$.

The objective of radar imaging is to reconstruct a *spatial* map of complex-valued reflection coefficient $\rho_{G}$ from the noisy backscattered signal $\tilde{s}$. This is an ill-posed linear inverse problem because the number of received signal samples is often much smaller than the number of pixels in the reflectivity map (i.e., M≪N). However, since the illuminated target typically only consists of a limited number of dominant scatterers, the reflection vector $\rho_{G}$ is *sparse*, i.e., containing a small number of nonzero entries. As a result, sparse reconstruction algorithms can be exploited to solve the underdetermined linear inverse problem (5).

The main principle of sparsity and compressive sensing is to find the sparsest solution of $\rho_{G}$ [4,5,6,7,8,9]:

(6) $$\min_{\rho_{G}}∥\rho_{G}{∥}_{0}\;\mathrm{subject}\;\mathrm{to}\;{∥\Phi_{G}\rho_{G}-\tilde{s}∥}_{2}\leq \epsilon ,$$

where ${∥\cdot ∥}_{0}$ denotes the $l_{0}$ norm, which returns the number of nonzero components of the argument, and ϵ≥0 is an error tolerance. However, this $l_{0}$-norm minimization formulation is NP-hard involving enumerative search and thus computationally intractable for practical applications. Extensive research studies have been conducted over the last two decades to seek more computationally tractable methods for solving sparse representation problems. Sparse reconstruction techniques in the literature can be categorized into five main groups [6]: (i) convex relaxation, (ii) greedy pursuit, (iii) Bayesian framework, (iv) nonconvex optimization, and (v) brute force. Interested readers are referred to [4,5,6,7] for comprehensive reviews on the state of the art of sparsity and compressive sensing.

## 3. The Blurring Problem in Conventional Sparsity-Driven Image Reconstruction

The linear signal model (5) is only strictly valid for the case of on-grid scatterers for which the true scatterers constituting the target are located exactly on the grid of the dictionary. Scatterers in real targets are however almost always off-grid; the imaging problem under consideration is no longer a linear inverse problem given in Equation (5) in the strict sense due to dictionary mismatch.

To formulate the problem, we consider a rotating target with *L* dominant scatterers with reflection coefficients $\rho_{O,l}$ and off-grid positions $p_{O,l}={\left[x_{O,l},y_{O,l}\right]}^{T}$ for l=1,⋯,L. The noise-free signal backscattered from the target is given by

(7) $$s=\sum_{l=1}^{L}\rho_{O,l}\vartheta \left(p_{O,l}\right)=\Phi_{O}\rho_{O},$$

where

(8a) $$\begin{array}{cc} \Phi_{O} & ={\left[\cdots ,\vartheta \left(p_{O,l}\right),\cdots \right]}_{l=1,\ldots ,L}, \end{array}$$

(8b) $$\begin{array}{cc} \rho_{O} & ={\left[\cdots ,\rho_{O,l},\cdots \right]}_{l=1,\ldots ,L}^{T}. \end{array}$$

Since the true scatterers do not coincide with the dictionary grid nodes, we have $\left\{p_{O,l}\right\}\notin \left\{p_{G,n}\right\}$ and thus $\Phi_{O}$ is not a submatrix of $\Phi_{G}$. As a result, $\Phi_{O}\rho_{O}\neq \Phi_{G}\rho_{G}$ and thus the strict equality in Equation (5) does not generally hold. Instead, we only have the approximation of $s\approx \Phi_{G}\rho_{G}$ for a sufficiently dense dictionary grid, thus leading to a sparse approximation problem:

(9) $$\tilde{s}\approx \Phi_{G}\rho_{G}+e.$$

As each true off-grid scatterer induces several on-grid atoms around its vicinity due to dictionary mismatch, the number of non-zero elements of $\rho_{G}$ is usually much larger than the number of true scatterers. In addition, the coefficient values of these on-grid atoms may vary depending on their distances to the corresponding true scatterers. Therefore, the coefficient vector $\rho_{G}$ is compressible rather than sparse, and its elements decay rapidly when sorted in order of decreasing magnitude. In practice, the sparse approximation problem is usually more challenging to solve than the sparse representation problem [6].

More importantly, the objective of the radar imaging problem under consideration does not directly align with the objective of the sparse approximation problem conventionally studied in the literature. Conventional sparse approximation algorithms generally aim to approximate a given signal with the lowest sparsity (least number of atoms), emphasising the approximation accuracy of the reconstructed signal $\hat{s}$ with respect to the original signal s in the *time* domain, while the solution for $\rho_{G}$ (i.e., which atoms in the dictionary are used to construct $\hat{s}$) is not the main focus. On the other hand, the objective of the considered radar imaging problem is to reconstruct a *spatial* map of $\rho_{G}$ of the target and thus the accuracy of the solution for $\rho_{G}$ is paramount. Therefore, solving for $\rho_{G}$ accurately using sparse approximation becomes a challenge in off-grid scatterers.

To illustrate the challenges associated with off-grid scatterers, we now present some simulation results for reconstructing the image of a rotating target consisting of 12 off-grid scatterers using sparse reconstruction. For demonstration purposes, we only show the reconstructed images obtained by Orthogonal Matching Pursuit (OMP) [44], a greedy pursuit technique, and least absolute shrinkage and selection operator (LASSO) [45], a convex relaxation technique. Similar observations were obtained by using other sparse reconstruction techniques whose results are omitted here for brevity.

**Figure 2:**
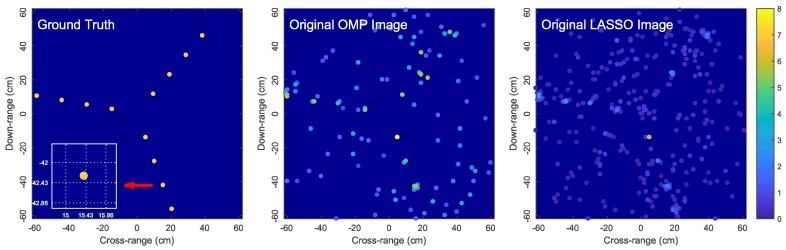
Problems with imaging off-grid scatterers: simulations with OMP and LASSO for a rotating target with 12 off-grid scatterers. The dictionary $\Phi_{G}$ is constructed from a regularly-spaced grid with $x_{G,n}\in \left\{-25\lambda :\lambda /2:25\lambda \right\}$ and $y_{G,n}\in \left\{-25\lambda :\lambda /2:25\lambda \right\}$. LASSO is halted when it achieves the same error residual as OMP.

**Figure 3:**
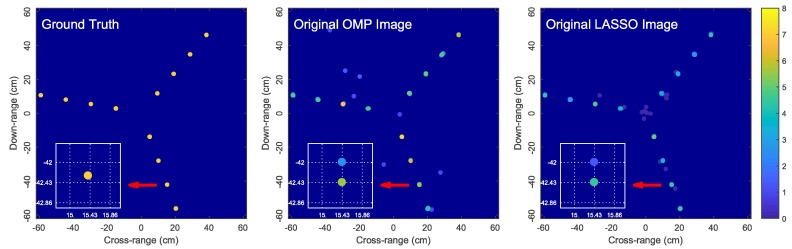
Reconstructed images obtained by OMP and LASSO for the same simulation setup as in Figure 2 but with the grid step reduced to λ/7, i.e., $x_{G,n}\in \left\{-25\lambda :\lambda /7:25\lambda \right\}$ and $y_{G,n}\in \left\{-25\lambda :\lambda /7:25\lambda \right\}$. The zoomed-in images show the locations of the solution atoms relative to a true scatterer (ground truth); here, dictionary grid points are located at intersections of dotted lines.

Figure 2 shows the OMP and LASSO images when the dictionary is constructed from a regularly-spaced grid with a grid step of λ/2. We observe that the reconstructed images are unsatisfactorily noisy and completely distorted with numerous spurious scatterers. This example demonstrates that a large mismatch between the locations of true scatterers and the hypothetical scatterers in the dictionary grid can significantly affect the imaging performance. Figure 3 shows the simulation results for the same simulation setup as in Figure 2 but with the grid step reduced to λ/7. More satisfactory images are obtained since the dictionary mismatch is reduced. However, since the true scatterers are not located on the dictionary grid, each true scatterer is approximated by a group of hypothetical scatterers of the dictionary grid (i.e., on-grid atoms) located in the surrounding vicinity of the true scatterer. For this reason, the signal energy of each true scatterer is spread over these on-grid atoms and thus the estimated reflection coefficients corresponding to these on-grid atoms are much lower than the reflection coefficient of the true scatterer. Consequently, the reconstructed images are blurred and scattered compared with the true image as illustrated in Figure 3.

## 4. Proposed Image Focusing Algorithm

In this section, we propose a new image focusing method to focus blurred sparsity-driven reconstructed images of rotating targets. It should be noted that the proposed method is applicable to images that are produced by any sparse reconstruction algorithms (not just limited to the OMP and LASSO images used for demonstration purposes in Section 3). The proposed image focusing method is composed of two stages: (I) atom clustering and (II) joint estimation of scatterer parameters. The details of each stage are presented as follows.

### 4.1. Stage I—Atom Clustering

**Figure 4:**
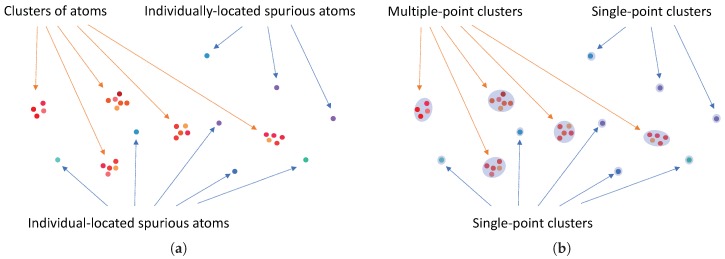
Example illustration of input and output for Stage I: (**a**) input of Stage I (before performing cluster analysis); (**b**) output of Stage I (after performing cluster analysis).

In the reconstructed image obtained by a sparse reconstruction algorithm, each true off-grid scatterer typically induces a group of on-grid atoms in the surrounding vicinity of the scatterer as a result of the dictionary mismatch as discussed in Section 3. In other words, the reconstructed image effectively contains several clusters of on-grid atoms corresponding to the true scatterers. Motivated by such a clustering behavior of the atoms obtained by the sparse reconstruction algorithm, we perform a cluster analysis to partition the atoms into a number of clusters as depicted in Figure 4. They can be either *multiple-point* clusters (which are formed by two or more atoms) or *single-point* clusters (which are formed by a single atom). A multiple-point cluster is likely to be a *genuine* cluster that corresponds to a true scatterer, while a single-point cluster is likely to be a *spurious* cluster associated with a spurious atom. However, there is still a possibility that some multiple-point clusters can be spurious clusters while some single-point clusters can be genuine. A discussion on how to handle the spurious atoms/clusters will be given at the end of Section 4.2.

Data clustering, also known as cluster analysis, has a long and rich history in a wide range of scientific fields. Interested readers are referred to [46,47,48] and the reference therein for detailed discussion and literature review on cluster analysis. Although various clustering techniques can be applied to perform atom clustering, we employ the *K*-means algorithm in this paper because of its simplicity and ease of implementation as well as its efficiency and empirical success as demonstrated in the literature.

Given *H* atoms obtained by the sparse reconstruction algorithm at locations $p_{h}$, h=1,⋯,H, we aim to cluster them into a set of *K* clusters $C_{k}$, k=1,⋯,K. The objective of the *K*-means algorithm is to determine a partition so that it minimizes the sum of squares of distances between the atoms and the corresponding cluster centroids. Specifically, with $\mu_{k}$ denoting the centroid coordinate of the cluster $C_{k}$, the objective function of the *K*-means algorithm to be minimized is given by

(10) $$f\left(C_{1},\cdots ,C_{K}\right)=\sum_{k=1}^{K}\sum_{p_{h}\in C_{k}}{∥p_{h}-\mu_{k}∥}^{2},$$

which is known to be an NP-hard problem [47]. The *K*-means algorithm minimizes this objective function by starting with a random partition, and iteratively reassigning each atom to its closest centroid and recomputing new cluster centroids. The common convergence criteria for *K*-means clustering include (i) no or minimal reassignment of data points to new cluster centroids, and (ii) no or minimal decrease in the objective function. *K*-means clustering is a greedy algorithm that may converge to a local minimum, although it has been shown in [48] that *K*-means clustering will converge to the global optimum with a high probability if clusters are well-separated. Therefore, different initial partitions may lead to different clustering results. To overcome this problem, the *K*-means algorithm is usually performed repeatedly using different initializations and the clustering result yielding the smallest value of the objective function is selected.

The *K*-means algorithm requires the number of clusters *K* as its input parameter. However, this information is unknown for our radar imaging application. Therefore, *K* has to be estimated. To this end, we perform the *K*-means algorithm for various values of *K* starting with K=1 and increasing *K* until the radius of the largest cluster reduces and falls below a preset threshold. Here, the radius of a cluster is defined as the distance from the centroids to the farthest point in the cluster. The preset threshold for cluster radius should be large enough to include all appropriate atoms clustered around the true scatterers to form the genuine clusters associated with the true scatterers while being small enough to exclude spurious atoms from these genuine clusters. Choosing suitable threshold values depends on the density (i.e., the grid step size) of the dictionary, as well as the noise level.

### 4.2. Stage II—Joint Estimation of Scatterer Parameters

**Figure 5:**
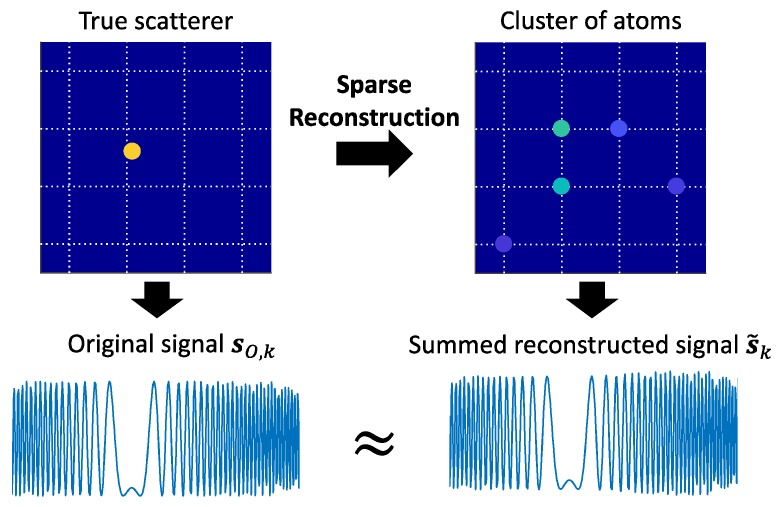
Approximate equivalence between the backscattered signal of a true scatterer and the summed reconstructed signal of the corresponding cluster of atoms.

Consider a cluster $C_{k}$ obtained from Stage I consisting of $U_{k}$ atom members with $p_{k,u}={\left[x_{k,u},y_{k,u}\right]}^{T}$ and ${\hat{\rho }}_{k,u}$ ($u\in \{1,2,\cdots ,U_{k}\}$) denoting the position and reflection coefficient of the *u*-th atom member. We assume that the cluster $C_{k}$ is genuine and corresponds to a true scatterer with unknown position $p_{O,k}={\left[x_{O,k},y_{O,k}\right]}^{T}$ and reflection coefficient $\rho_{O,k}$. The summed reconstructed backscattered signal ${\tilde{s}}_{k}$ calculated from all the atom members in the cluster is an estimate of the actual backscattered signal $s_{O,k}$ of the true scatterer (as depicted in Figure 5):

(11) $${\tilde{s}}_{k}\approx s_{O,k},$$

where

(12a) $$\begin{array}{cc} {\tilde{s}}_{k} & =\sum_{u=1}^{U_{k}}{\hat{\rho }}_{k,u}\vartheta \left(p_{k,u}\right), \end{array}$$

(12b) $$\begin{array}{cc} s_{O,k} & =\rho_{O,k}\vartheta \left(p_{O,k}\right). \end{array}$$

Motivated by this, we now consider an inverse problem aimed at jointly estimating the location $p_{O,k}$ and reflection coefficient $\rho_{O,k}$ of the true scatterer from the summed reconstructed backscattered signal ${\tilde{s}}_{k}$ via the least-squares criterion:

(13) $$\underset{\left\{\rho_{O,k},p_{O,k}\right\}}{\mathrm{minimize}}{∥{\tilde{s}}_{k}-\rho_{O,k}\vartheta \left(p_{O,k}\right)∥}_{2}.$$

This least-squares minimization is equivalent to

(14) $$\underset{\left\{\rho_{O,k},p_{O,k}\right\}}{\mathrm{minimize}}{∥\left[\begin{array}{c} \mathrm{Real}\{{\tilde{s}}_{k}\}\\ \mathrm{Imag}\{{\tilde{s}}_{k}\} \end{array}\right]-\left[\begin{array}{c} \mathrm{Real}\left\{\rho_{O,k}\vartheta \left(p_{O,k}\right)\right\}\\ \mathrm{Imag}\left\{\rho_{O,k}\vartheta \left(p_{O,k}\right)\right\} \end{array}\right]∥}_{2},$$

where

(15a) $$\begin{array}{cc} \mathrm{Real}\left\{\rho_{O,k}\vartheta \left(p_{O,k}\right)\right\} & ={\left[\cdots ,A\left(\mathrm{Real}\left\{\rho_{O,k}\right\}\cos\Phi_{O,k,m}-\mathrm{Imag}\left\{\rho_{O,k}\right\}\sin\Phi_{O,k,m}\right),\cdots \right]}_{m=1,\cdots ,M,}^{T} \end{array}$$

(15b) $$\begin{array}{cc} \mathrm{Imag}\left\{\rho_{O,k}\vartheta \left(p_{O,k}\right)\right\} & ={\left[\cdots ,A\left(\mathrm{Real}\left\{\rho_{O,k}\right\}\sin\Phi_{O,k,m}+\mathrm{Imag}\left\{\rho_{O,k}\right\}\cos\Phi_{O,k,m}\right),\cdots \right]}_{m=1,\cdots ,M,}^{T} \end{array}$$

(15c) $$\begin{array}{cc} \Phi_{O,k,m} & =b\;(x_{O,k}\sin\left(\Omega t_{m}\right)+y_{O,k}\cos\left(\Omega t_{m}\right)). \end{array}$$

We now let

(16) $$\begin{array}{cc} {\tilde{z}}_{k} & ={\left[\mathrm{Real}{\left\{{\tilde{s}}_{k}\right\}}^{T},\mathrm{Imag}{\left\{{\tilde{s}}_{k}\right\}}^{T}\right]}^{T}, \end{array}$$

(17) $$\begin{array}{cc} z_{O,k} & ={\left[\mathrm{Real}{\left\{\rho_{O,k}\vartheta \left(p_{O,k}\right)\right\}}^{T},\mathrm{Imag}{\left\{\rho_{O,k}\vartheta \left(p_{O,k}\right)\right\}}^{T}\right]}^{T}, \end{array}$$

and write $z_{O,k}\left(\xi_{O,k}\right)$ as an explicit function of

(18) $$\xi_{O,k}={\left[\rho_{O,k}^{R},\rho_{O,k}^{I},x_{O,k},y_{O,k}\right]}^{T},$$

with $\rho_{O,k}^{R}=\mathrm{Real}\left\{\rho_{O,k}\right\}$ and $\rho_{O,k}^{I}=\mathrm{Imag}\left\{\rho_{O,k}\right\}$. As a result, Equation (14) becomes

(19) $$\underset{\xi_{O,k}}{\mathrm{minimize}}{∥{\tilde{z}}_{O,k}-z_{O,k}\left(\xi_{O,k}\right)∥}_{2},$$

which is a least-squares estimation problem in the real-valued domain. This least-squares minimization is nonlinear and does not admit a closed-form solution. A numerical search algorithm can be obtained via iterative search approaches such as the steepest descent algorithm, the Nelder–Mead simplex algorithm, and the Gauss–Newton (GN) algorithm. The GN algorithm for solving Equation (19) is given by the following iteration [49]:

(20) $${\hat{\xi }}_{O,k}\left(j+1\right)={\hat{\xi }}_{O,k}\left(j\right)+{\left(J_{k}^{T}\left(j\right)J_{k}\left(j\right)\right)}^{-1}J_{k}^{T}\left(j\right)\left({\tilde{z}}_{k}-z_{O,k}\left({\hat{\xi }}_{O,k}\left(j\right)\right)\right)$$

for j=0,1,⋯. Here, $J_{k}\left(j\right)=J_{k}\left({\hat{\xi }}_{k}\left(j\right)\right)$ is the Jacobian matrix of $z_{O,k}$ with respect to $\xi_{O,k}$ evaluated at $\xi_{O,k}={\hat{\xi }}_{O,k}\left(j\right)$. The Jacobian matrix $J_{k}$ is given by

(21) $$J_{k}\left(\xi_{O,k}\right)={\left[J_{R,k}^{T}\left(\xi_{O,k}\right),J_{I,k}^{T}\left(\xi_{O,k}\right)\right]}^{T},$$

where

(22a) $$\begin{array}{cc} J_{R,k}\left(\xi_{O,k}\right) & ={\left[\cdots ,J_{R,k,m}^{T}\left(\xi_{O,k}\right),\cdots \right]}_{m=1,\cdots ,M,}^{T} \end{array}$$

(22b) $$\begin{array}{cc} J_{I,k}\left(\xi_{O,k}\right) & ={\left[\cdots ,J_{I,k,m}^{T}\left(\xi_{O,k}\right),\cdots \right]}_{m=1,\cdots ,M.}^{T} \end{array}$$

The expressions of $J_{R,k,m}\left(\xi_{O,k}\right)$ and $J_{I,k,m}\left(\xi_{O,k}\right)$ are

(23a) $$\begin{array}{cc} J_{R,k,m}\left(\xi_{O,k}\right) & =\left[J_{R,k,m}^{\left(1\right)},J_{R,k,m}^{\left(2\right)},J_{R,k,m}^{\left(3\right)},J_{R,k,m}^{\left(4\right)}\right], \end{array}$$

(23b) $$\begin{array}{cc} J_{I,k,m}\left(\xi_{O,k}\right) & =\left[J_{I,k,m}^{\left(1\right)},J_{I,k,m}^{\left(2\right)},J_{I,k,m}^{\left(3\right)},J_{I,k,m}^{\left(4\right)}\right], \end{array}$$

where

(24a) $$\begin{array}{cc} J_{R,k,m}^{\left(1\right)} & =A\cos\Phi_{O,k,m},\;J_{R,k,m}^{\left(2\right)}=-A\sin\Phi_{O,k,m}, \end{array}$$

(24b) $$\begin{array}{cc} J_{R,k,m}^{\left(3\right)} & =-Ab\sin\left(\Omega t_{m}\right)\left(\;\rho_{O,k}^{R}\sin\Phi_{O,k,m}\;+\;\rho_{O,k}^{I}\cos\Phi_{O,k,m}\;\right), \end{array}$$

(24c) $$\begin{array}{cc} J_{R,k,m}^{\left(4\right)} & =-Ab\cos\left(\Omega t_{m}\right)\left(\;\rho_{O,k}^{R}\sin\Phi_{O,k,m}\;+\;\rho_{O,k}^{I}\cos\Phi_{O,k,m}\;\right), \end{array}$$

and

(25a) $$\begin{array}{cc} J_{I,k,m}^{\left(1\right)} & =A\sin\Phi_{O,k,m},\;J_{I,k,m}^{\left(2\right)}=A\cos\Phi_{O,k,m}, \end{array}$$

(25b) $$\begin{array}{cc} J_{I,k,m}^{\left(3\right)} & =Ab\sin\left(\Omega t_{m}\right)\left(\rho_{O,k}^{R}\cos\Phi_{O,k,m}-\rho_{O,k}^{I}\sin\Phi_{O,k,m}\right), \end{array}$$

(25c) $$\begin{array}{cc} J_{I,k,m}^{\left(4\right)} & =Ab\cos\left(\Omega t_{m}\right)\left(\rho_{O,k}^{R}\cos\Phi_{O,k,m}-\rho_{O,k}^{I}\sin\Phi_{O,k,m}\right). \end{array}$$

To initialize the GN iteration (20), we use the energy-weighted center of the cluster $C_{k}$ as the initial position estimate:

(26) $${\hat{x}}_{O,k}\left(0\right)=\frac{\sum_{u=1}^{U_{k}}{\left|{\hat{\rho }}_{k,u}\right|}^{2}x_{k,u}}{\sum_{u=1}^{U_{k}}{\left|{\hat{\rho }}_{k,u}\right|}^{2}},\;{\hat{y}}_{O,k}\left(0\right)=\frac{\sum_{u=1}^{U_{k}}{\left|{\hat{\rho }}_{k,u}\right|}^{2}y_{k,u}}{\sum_{u=1}^{U_{k}}{\left|{\hat{\rho }}_{k,u}\right|}^{2}}$$

and the least-squares solution for $\rho_{O,k}$ based on the initial position estimate ${\hat{p}}_{O,k}\left(0\right)={\left[{\hat{x}}_{O,k}\left(0\right),{\hat{y}}_{O,k}\left(0\right)\right]}^{T}$ is used as the initial reflection estimate:

(27) $${\hat{\rho }}_{O,k}\left(0\right)={\left(g_{O,k}^{H}g_{O,k}\right)}^{-1}g_{O,k}^{H}{\tilde{z}}_{k},$$

where $g_{O,k}=\vartheta \left({\hat{p}}_{O,k}\left(0\right)\right)$. Here, the subscript ${}^{H}$ stands for the Hermitian transpose operation. The initial estimate of ξ for the GN iteration is

(28) $${\hat{\xi }}_{O,k}\left(0\right)\;=\;{\left[\mathrm{Real}\left\{{\hat{\rho }}_{O,k}\left(0\right)\right\},\mathrm{Imag}\left\{{\hat{\rho }}_{O,k}\left(0\right)\right\},{\hat{x}}_{O,k}\left(0\right),{\hat{y}}_{O,k}\left(0\right)\right]}^{T},$$

which is sufficiently close to the true solution of $\xi_{O,k}$, thus ensuring the convergence of the GN algorithm. For the radar imaging problem under consideration, we observe that the convergence of the GN algorithm can be achieved using 10–20 iterations. In general, the number of iterations for which the GN algorithm converges can be determined by examining the $l_{2}$ norm of the relative change of the estimate ${\hat{\xi }}_{O,k}$ over two consecutive iterations.

From the GN solution ${\hat{\xi }}_{O,k}^{\mathrm{GN}}={\hat{\xi }}_{O,k}\left(j_{\mathrm{final}}\right)$, we can extract the scatterer position estimate ${\hat{p}}_{O,k}^{\mathrm{GN}}={\left[{\hat{\xi }}_{O,k}^{\mathrm{GN}}\left(3\right),{\hat{\xi }}_{O,k}^{\mathrm{GN}}\left(4\right)\right]}^{T}$ and the scatterer reflection estimate ${\hat{\rho }}_{O,k}^{\mathrm{GN}}={\hat{\xi }}_{O,k}^{\mathrm{GN}}\left(1\right)+i\;{\hat{\xi }}_{O,k}^{\mathrm{GN}}\left(2\right)$. The cluster $C_{k}$ can now be replaced by an estimated scatterer with position ${\hat{p}}_{O,k}^{\mathrm{GN}}$ and reflection coefficient ${\hat{\rho }}_{O,k}^{\mathrm{GN}}$. In terms of imaging, this estimated scatterer produces a more physically meaningful and accurate representation of the true scatterer than a cluster of on-grid atoms in the surrounding vicinity of the scatterer.

The procedure for Stage II is summarized in Table 1. In the first step, each multiple-point cluster is replaced by an equivalent scatterer with the estimated location and reflection coefficient obtained by the GN algorithm. If the multiple-point cluster is a genuine cluster, the location and reflection coefficient of the equivalent scatterer is the estimate of the true location and coefficient of the corresponding true scatterer. In contrast, if the multiple-point cluster is a spurious cluster, it is replaced by an equivalent spurious scatterer. Note that, if a single-point cluster is a genuine cluster, the corresponding true scatterer must be located very close to a grid point in the dictionary. In this case, the true scatterer is readily estimated by the sole atom within the cluster, and we simply set the GN solutions to ${\hat{p}}_{O,k}^{\mathrm{GN}}=p_{k,1}$ and ${\hat{\rho }}_{O,k}^{\mathrm{GN}}={\hat{\rho }}_{k,1},$ where $p_{k,1}$ and ${\hat{\rho }}_{k,1}$ are the position and reflection coefficient of the atom. At the end of first step, we have a collection of *K* estimated scatterers. In the second step, a least-squares estimation is performed over these *K* atoms to re-calculate their reflection values to further improve the accuracy of reflection estimates.

## 5. Cramér–Rao Lower Bound for Scatterer Parameter Estimation

The radar imaging problem can be viewed as a parameter estimation problem for the locations and reflection coefficients of the scatterers constituting the target. Recall from Equation (7) that the noise-free backscattered signal $s=\sum_{l=1}^{L}\rho_{O,l}\vartheta \left(p_{O,l}\right)$ is a function of the scatterer positions $p_{O,l}={\left[x_{O,l},y_{O,l}\right]}^{T}$ and the scatterer coefficients $\rho_{O,l}$ (l=1,⋯,L). By decoupling the complex-valued coefficients into their real and imaginary parts (i.e., $\rho_{O,l}\to \left\{\rho_{O,l}^{R},\rho_{O,l}^{I}\right\}$) and converting the complex-valued signal model in Equation (7) into a real-valued model as

(29a) $$\begin{array}{cc} \tilde{s}\to \tilde{h} & ={\left[\mathrm{Real}{\left\{\tilde{s}\right\}}^{T},\mathrm{Imag}{\left\{\tilde{s}\right\}}^{T}\right]}^{T}, \end{array}$$

(29b) $$\begin{array}{cc} s\to h & ={\left[\mathrm{Real}{\left\{s\right\}}^{T},\mathrm{Imag}{\left\{s\right\}}^{T}\right]}^{T}, \end{array}$$

(29c) $$\begin{array}{cc} e\to \varepsilon & ={\left[\mathrm{Real}{\left\{e\right\}}^{T},\mathrm{Imag}{\left\{e\right\}}^{T}\right]}^{T}, \end{array}$$

we obtain

(30) $$\tilde{h}=h+\varepsilon .$$

The parameter estimation problem is stated as estimating $\Xi ={\left[\cdots ,\xi_{O,l}^{T},\cdots \right]}_{l=1,\cdots ,L}^{T}$ (where $\xi_{O,l}={\left[\rho_{O,l}^{R},\rho_{O,l}^{I},x_{O,l},y_{O,l}\right]}^{T}$) from the noisy nonlinear observation $\tilde{h}=h\left(\Xi \right)+\varepsilon$. In this context, we can derive the CRLB (i.e., the theoretical bound on the error variance) for the estimate of Ξ. For the purpose of computing the CRLB, we assume that the number of scatterers *L* is known. Noting that ε is an i.i.d. Gaussian noise vector, the CRLB for the estimate of Ξ is given by

(31) $$C_{\Xi }={\left(J_{\Xi }^{T}\Sigma_{\varepsilon }^{-1}J_{\Xi }\right)}^{-1},$$

where $\Sigma_{\varepsilon }=\frac{\sigma^{2}}{2}I_{2M\times 2M}$ ($I_{2M\times 2M}$ is the 2M×2M identity matrix) and $J_{\Xi }$ is the Jacobian matrix of h(Ξ) with respect to Ξ evaluated at the true parameter value. The expression of $J_{\Xi }$ is given by

(32a) $$\begin{array}{cc} J_{\Xi } & ={\left[J_{R,\Xi }^{T},J_{I,\Xi }^{T}\right]}^{T}, \end{array}$$

(32b) $$\begin{array}{cc} J_{R,\Xi } & ={\left[\cdots ,J_{R,\Xi ,m}^{T},\cdots \right]}_{m=1,\cdots ,M,}^{T} \end{array}$$

(32c) $$\begin{array}{cc} J_{I,\Xi } & ={\left[\cdots ,J_{I,\Xi ,m}^{T},\cdots \right]}_{m=1,\cdots ,M,}^{T} \end{array}$$

(32d) $$\begin{array}{cc} J_{R,\Xi ,m} & ={\left[\cdots ,J_{R,\Xi ,m,l}\left(\xi_{O,l}\right),\cdots \right]}_{l=1,\cdots ,L,} \end{array}$$

(32e) $$\begin{array}{cc} J_{I,\Xi ,m} & ={\left[\cdots ,J_{I,\Xi ,m,l}\left(\xi_{O,l}\right),\cdots \right]}_{l=1,\cdots ,L,} \end{array}$$

where $J_{R,\Xi ,m,l}\left(\xi_{O,l}\right)$ and $J_{I,\Xi ,m,l}\left(\xi_{O,l}\right)$ have the same expressions as $J_{R,k,m}\left(\xi_{O,k}\right)$ and $J_{I,k,m}\left(\xi_{O,k}\right)$ in Equation (23), respectively, except replacing $\xi_{O,k}$ with $\xi_{O,l}$.

## 6. Simulations

### 6.1. Simulation Setup

We consider a synthetic 2D radar imaging scenario where a monostatic single-frequency continuous-wave radar operating at a frequency of 10 GHz (λ=0.03 m) illuminates a far-field target rotating around the origin of its local coordinates at Ω=31.4159 rad/s. The continuous-time backscattered signal is sampled at the rate of 6 kHz. The signal-to-noise ratio (SNR) is set to 10 dB. The total number of data samples is M=1200. The dictionary $\Phi_{G}$ is constructed from a regularly spaced grid with $x_{G,n}\in \left\{-25\lambda :\lambda /7:25\lambda \right\}$ and $y_{G,n}\in \left\{-25\lambda :\lambda /7:25\lambda \right\}$. For demonstration purposes, images obtained by OMP, LASSO and Subspace Pursuit (SP) [50] are post-processed by the proposed algorithm, and a comparison of focused images is provided. In the simulations, OMP, LASSO and SP are stopped when the relative change in the norm of the signal residual is less than δ=0.005. Since the sparsity level of the true coefficient vector is unknown, we use the sparsity level of the OMP solution as the sparsity input for the SP algorithm. The radius threshold for cluster analysis in Stage I of the proposed algorithm is λ/2, and 20 GN iterations are used in Stage II.

**Figure 6:**
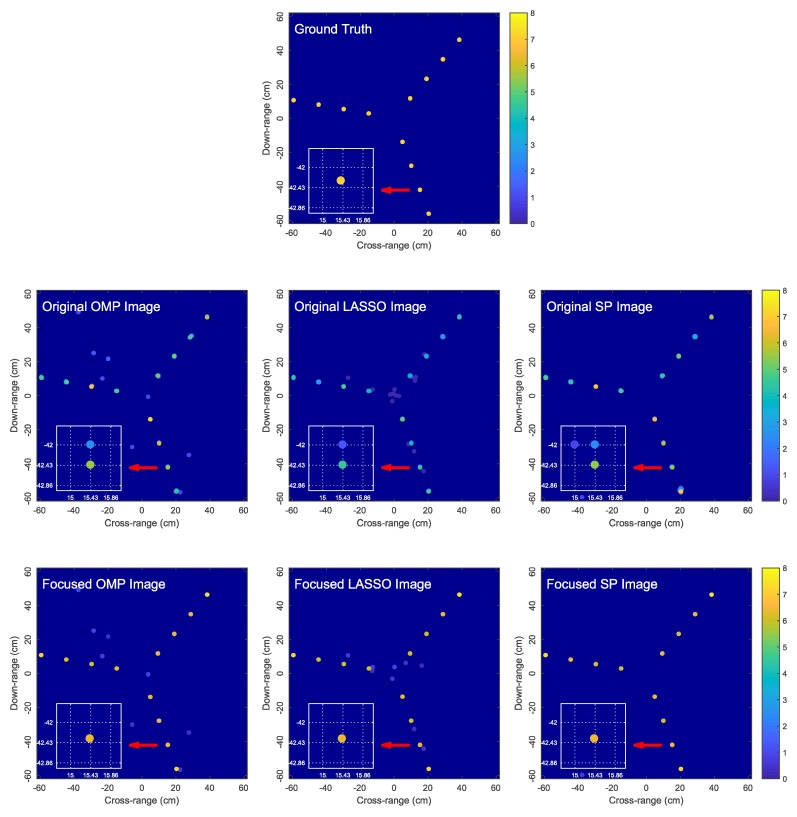
Comparison of the original OMP, LASSO and SP images and the corresponding focused images obtained by the proposed algorithm for Target 1. In the zoomed-in images, dictionary grid points are located at intersections of dotted lines.

**Figure 7:**
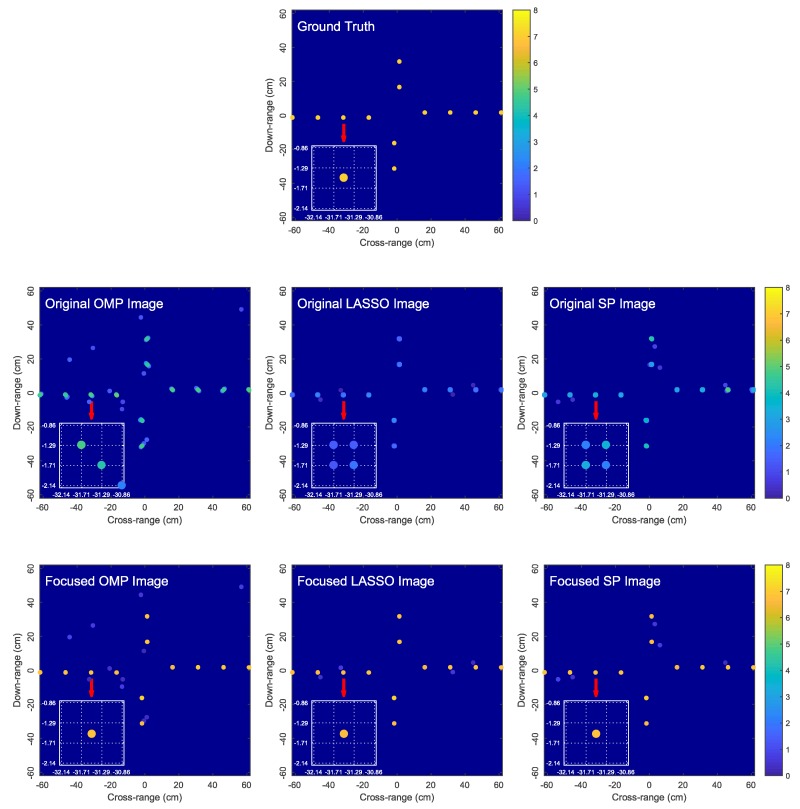
Comparison of the original OMP, LASSO and SP images and the corresponding focused images obtained by the proposed algorithm for Target 2. In the zoomed-in images, dictionary grid points are located at intersections of dotted lines.

Two target models are considered as shown in Figure 6 and Figure 7. Target 1 consists of 12 scatterers located at the bearing angles of $-70^{\circ }$, $50^{\circ }$ and $170^{\circ }$ with respect to the *x*-axis and the radial distances of 0.15, 0.30, 0.45 and 0.60 meters from the center of rotation ${\left[0,0\right]}^{T}$. Target 2 is made up of 12 scatterers, where six scatterers are located at ${\left[0.165,0.015\right]}^{T}$, ${\left[0.315,0.015\right]}^{T}$, ${\left[0.465,0.015\right]}^{T}$, ${\left[0.615,0.015\right]}^{T}$, ${\left[0.015,0.165\right]}^{T}$ and ${\left[0.015,0.315\right]}^{T}$ meters while the remaining six scatterers are the reflections of the first six scatterers over the origin. Target 2 represents a worst-case target model because each scatterer is midway between the dictionary grid points (i.e., offset from the grid points by one-half of the grid step size in both *x*- and *y*-axes). The true reflection coefficient of each scatterer is set to (5+5i).

### 6.2. Proposed Algorithm versus Conventional Algorithms

Figure 6 and Figure 7 compare the original OMP, LASSO and SP images and the corresponding focused images obtained by the proposed algorithm for two simulated target models. We observe that OMP, LASSO and SP result in blurred and low-contrast reconstructed images due to the dictionary mismatch problem as explained in Section 3, where each true scatterer is represented by a cluster of atoms. The proposed algorithm is capable of effectively focusing the blurred OMP, LASSO and SP images by producing sharper images with each true scatterer accurately represented by a single estimated scatterer. Since only one estimated scatterer is used to represent a true scatterer, we not only avoid the image blurring problem associated with OMP, LASSO and SP, but also obtain a more accurate image intensity as a result of not spreading scatterer signal energy over multiple atoms. Moreover, the GN algorithm accurately estimates the locations and reflection coefficients of the true scatterers, producing a highly accurate image of the true target.

We also observed from Figure 6 and Figure 7 that, although the proposed focusing algorithm does not directly eliminate the spurious atoms given by the sparse reconstruction algorithm, the spurious atoms are weakened (i.e., their reflection coefficients are reduced) since the signal energy is now concentrated into the highly-accurate estimated scatterers. Given the distinction in the reflection coefficients between the genuine and spurious scatterers in the focused image, the spurious scatterers may be discarded using an additional thresholding step.

### 6.3. RMSE versus CRLB

We now compare the root-mean-squared-error (RMSE) of the location and reflection estimates obtained by the proposed algorithm with the square root of CRLB (RCRLB) derived in Section 5. The RMSEs for the position estimate and the reflection estimate, averaged over $J_{MC}=500$ Monte Carlo (MC) runs and averaged across over all the scatterers of the target, are defined as

$$\begin{array}{c} {\mathrm{RMSE}}_{\mathrm{position}}={\left(\frac{1}{LJ_{MC}}\sum_{l=1}^{L}\sum_{j=1}^{J_{MC}}{∥{\hat{p}}_{O,l}^{\left(j\right)}-p_{O,l}∥}_{2}^{2}\right)}^{1/2},\\ {\mathrm{RMSE}}_{\mathrm{reflection}}={\left(\frac{1}{LJ_{MC}}\sum_{l=1}^{L}\sum_{j=1}^{J_{MC}}{\left|{\hat{\rho }}_{O,l}^{\left(j\right)}-\rho_{O,l}\right|}^{2}\right)}^{1/2}, \end{array}$$

where ${\hat{p}}_{O,l}^{\left(j\right)}$ and ${\hat{\rho }}_{O,l}^{\left(j\right)}$ are the estimates of the position and reflection coefficient for the *l*-th scatterer at the *j*-th MC run. The theoretical lower bound for ${\mathrm{RMSE}}_{\mathrm{position}}$ and ${\mathrm{RMSE}}_{\mathrm{reflection}}$ are given by

$$\begin{array}{cc} {\mathrm{RCRLB}}_{\mathrm{position}} & ={\left(\frac{1}{L}\sum_{l=1}^{L}\left(C_{\Xi }\left(4l-1\right)+C_{\Xi }\left(4l\right)\right)\right)}^{1/2},\\ {\mathrm{RCRLB}}_{\mathrm{reflection}} & ={\left(\frac{1}{L}\sum_{l=1}^{L}\left(C_{\Xi }\left(4l-3\right)+C_{\Xi }\left(4l-2\right)\right)\right)}^{1/2}, \end{array}$$

where $C_{\Xi }\left(i\right)$ is the *i*-th diagonal element of $C_{\Xi }$.

Table 2 reports the RMSE performance versus RCRLB for Targets 1 and 2. We observe that the RMSE of the reflection estimate is very close to the theoretical RCRLB, especially for high SNR values, while the RMSE of the position estimate (i.e., about 7 to 14 times smaller than the dictionary grid step) is comparable to the theoretical RCRLB. This confirms the capability of the proposed algorithm to produce high-accuracy radar images for rotating targets.

### 6.4. Non-Centrosymmetric Target

**Figure 8:**
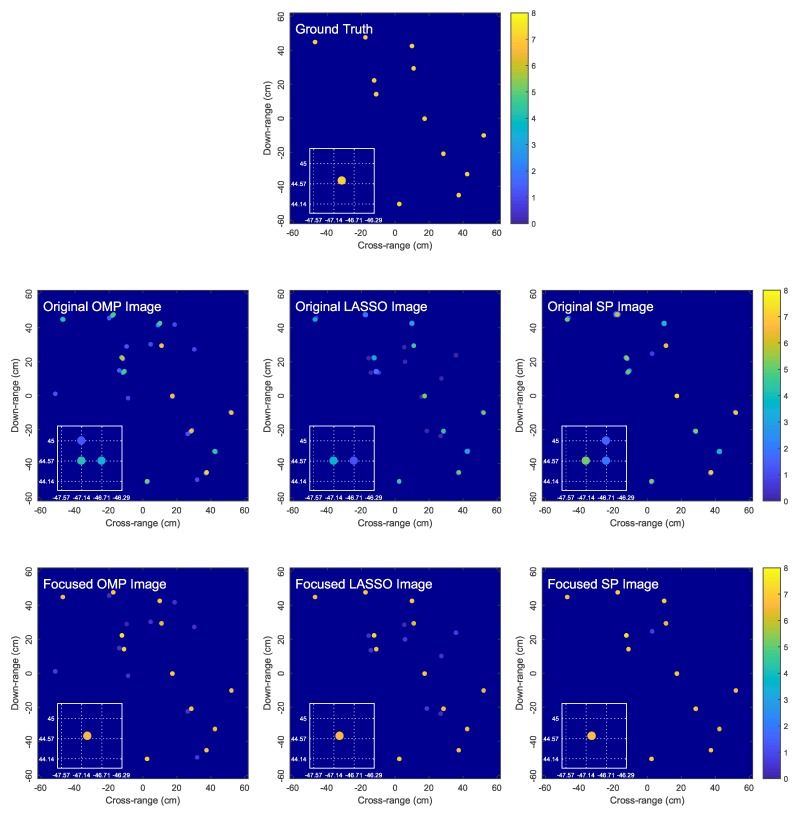
Comparison of the original OMP, LASSO and SP images and the corresponding focused images obtained by the proposed algorithm for a non-centrosymmetric target with 12 randomly-located scatterers. In the zoomed-in images, dictionary grid points are located at intersections of dotted lines.

The proposed algorithm is applicable to non-centrosymmetric targets. Figure 8 shows the original OMP, LASSO and SP images, as well as their corresponding focused images obtained by the proposed algorithm for a non-centrosymmetric target with 12 randomly-located scatterers. Consistent with Figure 6 and Figure 7, the results in Figure 8 clearly confirm the effectiveness of the proposed image focusing algorithm.

### 6.5. Runtime Performance

For complexity comparison purposes, Table 3 compares the sparse-reconstruction runtimes of OMP, LASSO and SP with the corresponding post-processing runtimes of the proposed imaging focusing algorithm (implemented in MATLAB (R2017a, The MathWorks, Natick, MA, USA)) and executed on the same hardware platform). We observe that the proposed image focusing algorithm consumes much less runtime than the main sparse-reconstruction algorithms (only 4.65%, 1.82% and 18.54% of the total runtime for OMP, LASSO and SP, respectively). Therefore, the proposed algorithm only requires a relatively small computational overhead.

### 6.6. Scatterer Separation Test

In this simulation, we consider two closely-spaced scatterers and numerically examine the probability of successful separation for various spacings between them. The first scatterer is fixed at a location while the location of the second scatterer is varied. Here, the SNR is set to 5 dB. For each tested location of the second scatterer, we perform a simulation for 500 MC runs. In each run, the two scatterers are considered to be successfully separated if the error norms of the scatterer position estimates are less than twice the grid step size and the error norms of the scatterer reflection estimates are less than 14.14% of the norms of the true coefficient values (roughly twice the reflection RCRLB value for SNR = 5 dB in Table 2). Overall, the scatterers are considered to be separable if a separation success rate of more than 85% is achieved. In this simulation, OMP is used for initial sparse reconstruction, and the proposed algorithm is then executed to focus the OMP image.

**Figure 9:**
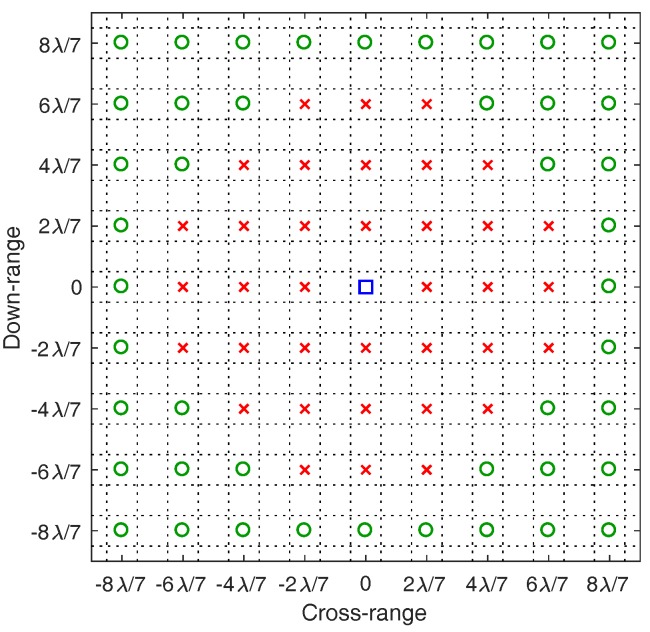
Result of the separation test: 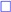
—the fixed location of the first scatterer, 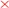
—the tested location of the second scatterer for which the two scatterers are not separable, 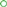
—the tested location of the second scatterer for which the two scatterers are separable. Dictionary grid points are located at intersections of dotted lines. Note that the coordinate origin in this plot is shifted to the location of the first scatterer. Identical results are obtained when the first scatterer is placed at the radial distance of 0.3 m or 0.6 m from the rotation center.

For a fair result, we consider a worst-case scenario where both the scatterers are located midway between the dictionary grids points (i.e., off-set from the grid points by a half of the grid step size in both the *x*- and *y*-axes). Figure 9 shows the result of the separation test. We observe that the minimum distance between the two scatterers for them to be separable is about λ. This is consistent with the fact that the value of λ/2 is used as the radius threshold for cluster analysis in Stage I. Note that the separation capability may be improved further by using a denser dictionary grid and thus a smaller value for the cluster radius threshold. However, this improvement comes at the expense of higher computational cost.

## 7. Conclusions

In this paper, we have developed a new image focusing algorithm to focus blurred sparse-reconstructed images of rotating targets in the general case of off-grid scatterers. The main reason for the blurring problem of the reconstructed images obtained by sparse reconstruction algorithms was shown to be the mismatch between the true off-grid scatterers constituting the target and the grid of the dictionary. To overcome such a problem, the proposed algorithm exploits cluster analysis and joint scatterer parameter estimation to focus the blurred sparsity-driven reconstructed images. Comparative simulation studies were carried out to demonstrate the effectiveness of the proposed algorithm in terms of image focusing at low computational overheads. In addition, the proposed algorithm was empirically shown to attain a mean-squared error performance comparable to the theoretical CRLB in terms of estimating the unknown parameters (i.e., locations and coefficients) of the true scatterers.

The proposed algorithm can be extended to other off-grid sparse estimation problems for which the atoms in the solution obtained by sparse reconstruction algorithms exhibit a clustering behavior around the true scatterers due to dictionary mismatch. Such a smearing effect (i.e., the clustering of atoms around the true scatterers) occurs in various sparsity-driven radar imaging problems, such as radar coincidence imaging [43] and synthetic aperture radar (SAR) imaging [51]. For different problems, a new derivation of the Jacobian matrix of the Gauss–Newton iteration in Stage II is required, while the overall structure of the proposed algorithm remains unchanged.

## Figures and Tables

**Table 1:** Computational steps of stage II.

**Step 1:** Replace clusters obtained in Stage I by equivalent scatterers **for** cluster k=1; k:=k+1; k≤K **do** **if**$U_{k}>1$ - compute the summed reconstructed backscattered signal ${\tilde{z}}_{k}$ as in Equation (12a) - estimate ${\hat{p}}_{O,k}^{\mathrm{GN}}$ and ${\hat{\rho }}_{O,k}^{\mathrm{GN}}$ via the GN iteration (20) **else** (i.e., single-point cluster) - set the GN solution to ${\hat{p}}_{O,k}^{\mathrm{GN}}=p_{k,1}$ and ${\hat{\rho }}_{O,k}^{\mathrm{GN}}={\hat{\rho }}_{k,1}$ **end if** **end for** **Step 2:** Re-estimate the reflection coefficients of obtained equivalent scatterers Construct new sub-dictionary matrix based on ${\hat{p}}_{O,k}^{\mathrm{GN}}$ $\Psi ={\left[\cdots ,\vartheta \left({\hat{p}}_{O,k}^{\mathrm{GN}}\right),\cdots \right]}_{k=1,\cdots ,K}$ Compute new reflection coefficients in least-squares sense: ${\left[{\hat{\hat{\rho }}}_{O,1},\cdots ,{\hat{\hat{\rho }}}_{O,K}\right]}^{T}={\left(\Psi^{H}\Psi \right)}^{-1}\Psi^{H}\tilde{s}$

**Table 2:** RMSE performance.

	Target 1							Target 2				
	Position (mm)			Reflection				Position (mm)			Reflection	
SNR (dB)	RMSE	RCRLB		RMSE	RCRLB			RMSE	RCRLB		RMSE	RCRLB
5	0.604	0.210		0.490	0.435			0.526	0.232		0.567	0.479
6	0.556	0.187		0.438	0.387			0.366	0.206		0.463	0.427
7	0.506	0.167		0.389	0.345			0.407	0.184		0.425	0.381
8	0.444	0.149		0.345	0.308			0.294	0.164		0.357	0.339
9	0.397	0.132		0.313	0.274			0.298	0.146		0.346	0.302
10	0.352	0.118		0.283	0.244			0.254	0.130		0.286	0.269

**Table 3:** Average runtime performance.

	OMP	LASSO	SP
Sparse-reconstruction runtime (s)	7.369	23.771	0.827
Post-processing runtime ${}^{†}$ (s)	0.359	0.440	0.188
Total runtime (s)	7.728	24.211	1.015

${}^{†}$ Runtime for performing the proposed image focusing algorithm.
